# Neuroprotective actions of 2,4-dinitrophenol: Friend or foe?

**DOI:** 10.1590/S1980-57642008DN10400002

**Published:** 2007

**Authors:** Sérgio Teixeira Ferreira, Fernanda Guarino De Felice

**Affiliations:** Instituto de Bioquímica Médica, Programa de Bioquímica e Biofísica Celular, Universidade Federal do Rio de Janeiro, Rio de Janeiro, RJ, Brazil.

**Keywords:** 2,4-dinitrophenol, amyloid-β peptide, neurodegenerative diseases, neuroprotection, oxidative stress, therapy, 2,4-dinitrofenol, peptídeo β-amilóide, doenças neurodegenerativas, neuroproteção, estresse oxidativo, terapia

## Abstract

2,4-dinitrophenol (DNP) has long been known to be toxic at high concentrations, an effect related to uncoupling of mitochondrial oxidative phosphorylation. Five years ago, however, we reported that low concentrations of DNP protect neurons against the toxicity of the amyloid-β peptide. Since then, a number of other studies have provided evidence of beneficial actions of DNP (at low concentrations), including neuroprotection against different types of insult, blockade of amyloid aggregation, stimulation of neurite outgrowth and neuronal differentiation, and even extension of lifespan in certain organisms. Some of these effects appear due to mild mitochondrial uncoupling and prevention of oxidative stress, whereas other actions are related to activation of additional intracellular signaling pathways. This study discusses the evidence supporting beneficial neuroprotective actions of DNP. DNP and other compounds with similar biological activities may be of interest in the development of novel therapeutic approaches for neurodegenerative diseases and other neurological disorders.

## Abstract (pt)

O 2,4-dinitrofenol (DNP) tem sido conhecido há bastante tempo como tóxico em altas concentrações, um efeito relacionado a desacoplamento da fosforilação oxidativa nas mitocôndrias. Há cinco anos, entretanto, nós relatamos que baixas concentrações do DNF protegem neurônios da toxicidade do peptídeo β-amilóide. Desde então, outros estudos trouxeram evidência adicional dos efeitos benéficos do DNP (em baixas concentrações), incluindo neuroproteção contra diferentes tipos de agressão, bloqueio da agregação do amilóide, estimulação de crescimento neurítico e diferenciação neuronal, e mesmo extensão da sobrevida em alguns organismos. Alguns desses efeitos parecem ser devidos a leve desacoplamento mitocondrial e prevenção do estresse oxidativo, enquanto outras ações são relacionadas à ativação de sistemas adicionais de sinalização intracelular. Este estudo discute a as evidências que dão suporte a ações neuroprotetoras benéficas do DNP. DNP e outros compostos com atividades biológicas similares podem ser de interesse no desenvolvimento de novas abordagens terapêuticas para doenças neurodegenerativas e outros transtornos neurológicos.

At high concentrations, *2,4-dinitrophenol* (DNP) and other nitrophenols are toxic to humans and animals. Toxicity is caused by interference with cellular energy metabolism due to uncoupling of oxidative phosphorylation. DNP allows the mitochondrial respiratory chain to proceed without producing ATP, thus increasing the basal metabolic rate. For this reason, during the 1930s DNP was used as a weight loss drug in the treatment of obesity.^1^ However, in 1938 use of DNP as a drug for human consumption was banned by the US Food and Drug Administration due to a number of deaths and to the development of cataracts amongst some users.

On the other hand, recent *in vitro* and *in vivo* studies have shown that DNP may have interesting beneficial effects at low concentrations, for example in preventing neuronal degeneration induced by different insults. The present work highlights some of the recent findings indicating beneficial actions of DNP at low doses. Based on its favorable properties, we suggest that DNP could be used as a lead compound for the development of novel therapeutic approaches for Alzheimer’s disease (AD) and other still untreatable neurodegenerative diseases as well as for some acute neurological disorders.

## Mild mitochondrial uncoupling and beneficial effects of DNP

Considerable evidence indicates that reactive oxygen species (ROS) are centrally implicated in a number of chronic and acute disorders. Of special interest is the case of several neurological diseases in which ROS participate in neuronal damage triggered by various types of insult. Among these conditions are Alzheimer’s, Parkinson’s and Huntington’s diseases, amyotrophic lateral sclerosis and AIDS dementia complex.^2,3^ Other acute insults leading to massive brain cell death that have been related to oxidative damage include hypoglycemia, neurological trauma, stroke, ischemia/reperfusion and epilepsy.^2,3^

The mitochondrial respiratory chain is the major source of intracellular ROS production under physiological and many pathological conditions.^4^ ROS formation results from monoelectronic reduction of oxygen, generating the superoxide anion instead of water, which is increased under conditions of high electrochemical gradient across the mitochondrial membrane.^4^ This suggests that mild mitochondrial uncoupling, with a corresponding decrease in electrochemical gradient, might prevent excessive ROS formation and cellular oxidative damage in certain pathological conditions.

It was recently demonstrated that rats treated intraperitoneally with DNP showed significant protection against brain damage caused by striatal injection of the N-methyl-D-aspartate (NMDA) receptor agonist quinolinic acid.^5^ Overactivation of NMDA receptors (by excessive glutamate or other agonists) leads to excitotoxic neuronal damage, involving increased intracellular calcium levels and increased mitrochondrial ROS production.^6^ Interestingly, rats administered DNP either 1 h before or 3 h following quinolinic acid infusion developed lesions that were 25% smaller than control animals.^5^ The mechanism of neuroprotection by DNP was subsequently found to be related to the decrease in mitochondrial Ca^2+^ levels and in ROS formation.^7^ These findings indicate that DNP confers protection against acute brain injury involving excitotoxic pathways by mechanisms that maintain mitochondrial function.

Another recent study showed that intraperitoneal administration of DNP reduces the infarct volume by approximately 40% in a model of focal ischemia-reperfusion injury in rat brains.^8^ Interestingly, overexpression of human uncoupling protein 2 (UCP-2) in mice has been shown to reduce brain damage after experimental stroke and traumatic brain injury,^9^ suggesting that mitochondrial uncoupling might play a role in the prevention of damage. The same study also showed that neuronal death induced by oxygen and glucose deprivation in cultured cortical neurons was reduced in the presence of DNP under conditions that caused mild mitochondrial uncoupling.

In a different experimental paradigm, intraperitoneal injection of DNP in rats was shown to improve mitochondrial function, attenuate oxidative damage and increase white matter sparing in the contused spinal cord,^10^ suggesting that this might constitute a novel approach for the treatment of spinal cord contusion damage. Taken together, these studies suggest that, contrary to the uncontrolled and deleterious mitochondrial uncoupling that takes place in the presence of high concentrations of DNP, mild uncoupling induced by low doses of DNP may actually be beneficial in terms of reducing oxidative neuronal damage in different pathological conditions.

Accumulating evidence indicates that aging is, at least in part, linked to changes in mitochondrial metabolism and ROS formation. In this regard, it has been proposed that, similar to caloric restriction (11), mild mitochondrial uncoupling could increase lifespan by reduction of oxidative stress (4). Although more studies are certainly warranted to determine whether this might be the case in higher organisms, it is interesting to note that DNP administration has recently been shown to prolong the lifespan of both Drosophila melanogaster and *Saccharomyces cerevisiae*.^12,13^

## Non-mitochondrial beneficial actions of DNP

### Alzheimer’s disease and other amyloidosis: DNP as an anti-amyloidogenic compound

Amyloid diseases comprise an important class of human pathologies, including Alzheimer’s and Parkinson’s diseases, the spongiform encephalopathies, type II diabetes mellitus and various forms of systemic amyloidosis. These diseases have in common the occurrence of lesions in affected tissues, consisting of intra- or extracellular aggregates of misfolded proteins that have been closely associated with degeneration. At present, more than 20 different proteins or peptides are known to form amyloid deposits associated with human pathologies.^14^

Alzheimer’s disease (AD) is the most common cause of progressive decline of cognitive function in aged humans. It is estimated that AD currently affects 17–25 million people worldwide. In Western countries, it represents the fourth leading cause of death after heart diseases, cancer and stroke.^15^ The combination of increasing prevalence together with the relatively long survival (7 years on average) after diagnosis of AD places a heavy social and economic burden on family members and society. In the United States alone, the annual direct and indirect economic costs associated with AD are estimated at US$80–100 billion.^16^

AD is neuropathologically characterized by extracellular accumulation of fibrillar amyloid beta peptide (Aβ in senile plaques, intraneuronal neurofibrillary tangles consisting of abnormally hyperphosphorylated tau protein, oxidative neuronal damage, synaptic degeneration and, more recently, by the build-up of soluble Aβ oligomers.^17-19^ Aggregation of Aβ appears to initiate a cascade of events that leads to neuronal dysfunction (likely explaining the early cognitive decline characteristic of AD) and, ultimately, to nerve cell death. In this regard, pharmacological intervention at initial steps of the amyloid aggregation process, e.g., preventing the formation of Aβ oligomers, could possibly halt or even reverse the pathogenesis of AD.

Structure-based drug design approaches to develop inhibitors of Aβ aggregation and neurotoxicity have been hampered by the lack of detailed structural information on either soluble, oligomeric or fibrillar aggregated Aβ. With the goal of developing mechanism-based strategies to interfere with amyloid aggregation and toxicity, we initially investigated the contribution of hydrophobic interactions to the stability of Aβ fibrils.^20^ These studies revealed two important aspects of amyloid aggregation:

1) pre-aggregated amyloid fibrils could be reversibly disaggregated by incubation at low temperatures, a signature of protein aggregates that are stabilized by hydrophobic interactions;
2) the stability of amyloid fibrils in denaturant solutions was markedly increased when fibrils were assembled from the Aβ_1-42_ or Aβ_1-43_ peptides compared to a C-terminally truncated peptide, Aβ_1-28_, which lacks a sequence of non-polar amino acids spanning residues 29-42 of Aβ.

These results suggested that hydrophobic interactions mediated by the C-terminal domain (residues 29-42) of Aβ are important for the stability of amyloid aggregates.

We thus hypothesized that low molecular weight hydrophobic compounds might destabilize and disaggregate amyloid aggregates. After examining a number of moderately hydrophobic compounds (i.e., sufficiently hydrophobic to interfere with amyloid aggregation but still maintaining good solubility in aqueous media), we found that low micromolar concentrations of DNP, 3-nitrophenol and other structurally related compounds completely prevented amyloid aggregation *in vitro* and caused the disassembly of pre-aggregated amyloid fibrils.^20^ Of significant interest, DNP and 3-nitrophenol completely blocked the neurotoxicity of Aβ to rat hippocampal neurons in primary culture, and caused a marked reduction in the area occupied by amyloid deposits in an acute model of amyloid deposition in rat brains.^20^ To our knowledge, this was the first published report showing a neuroprotective action of DNP. In a subsequent study, we demonstrated that DNP and other disubstituted aromatic compounds inhibit the formation of soluble oligomers of Aβ peptide, also known as “ADDLs” (an acronym for Aβ-*Derived Diffusible Ligands*), and completely block their toxicity to cortical and hippocampal neurons in culture.^21^

Recent evidence indicates that Aβ oligomers (ADDLs), rather than the mature amyloid fibrils present in the senile plaques, are the main neurotoxic species involved in the pathogenesis of AD.^18,19^ Thus, the ability of DNP and of other small-molecule compounds to block the formation of Aβ oligomers might constitute a promising pharmacological approach to interfere with an early event in the pathogenesis of AD. Considering that DNP exhibits a number of potentially adverse side-effects, the fact that other compounds we have characterized have also been shown to potently block Aβ oligomer formation and neurotoxicity^21^ appears particularly interesting in this regard.

Interestingly, we have recently found that DNP and other di-substituted aromatic compounds also inhibit the formation of amyloid fibrils from both wild type and variant forms of human lysozyme associated with hereditary systemic amyloidosis.^22^

Furthermore, DNP inhibits the aggregation of human transthyretin implicated in familial amyloidotic polyneuropathy and senile systemic amyloidosis.^23,24^ These findings suggest that the ability of DNP and related compounds to destabilize aberrant protein-protein interactions may find possible applications in other amyloid diseases in addition to AD.

### DNP induces neurite outgrowth and neuronal differentiation: Possible implications in neurological diseases

The cytoskeleton plays a key role in maintaining the highly asymmetrical cell shape and structural polarity that are essential for neuronal physiology. For example, cytoskeletal reorganization is required for neurite outgrowth and for synapse formation and remodeling. Several neurodegenerative diseases, including AD, Parkinson’s disease, multiple sclerosis, amyotrophic lateral sclerosis and Huntington’s disease are characterized by abnormal cytoskeleton assembly and, as a consequence, impairment of neurotransmission.^25^ This may partly explain the early cognitive loss and functional impairment observed in patients with neurodegenerative diseases. There are currently no clinically approved and effective treatments available to prevent neurodegeneration in these diseases. In this regard, an interesting new approach could be the use of compounds capable of preventing synapse and neurite degeneration and/or inducing neuritogenesis.

Neurite dystrophy is a characteristic pathological finding in AD brains.^26^ Morphologic studies have demonstrated significant loss of synaptic connectivity in many regions of the neocortex and hippocampus and a strong correlation between decreased synaptic density and cognitive decline in AD.^27^ We recently showed that low concentrations of DNP (≤20 βM) promote neurite outgrowth in cultured hippocampal and cortical neurons, and induce neuronal differentiation in a neuroblastoma cell line.^28^ Interestingly, we found that neurite outgrowth induced by DNP is accompanied by increased levels of tau protein and cAMP in both primary cortical and hippocampal neuronal cultures as well as in the N2A murine neuroblastoma cell line.^28^

Under physiological conditions, tau facilitates tubulin assembly,^29^ nucleates the polymerization and stabilizes microtubules.^30^ In CNS neurons, tau has been shown to be centrally involved in neurite outgrowth.^31^ By contrast, under pathological conditions (such as in Alzheimer’s disease and other tauopathies), tau undergoes abnormal hyperphosphorylation,^32^ resulting in self-aggregation and formation of insoluble cytoplasmic inclusions that appear to be related to the destabilization of microtubules.^31^ Therefore, the increase in neuronal tau levels induced by DNP may facilitate microtubule growth and/or stabilize existing microtubules, resulting in increased cellular capacity to extend neurites. An important observation was that the increase in neuronal tau levels induced by DNP was not accompanied by tau hyperphosphorylation,^28^ which, as noted above, has been shown to impair its function as a microtubule binding protein. Thus, one of the mechanisms by which DNP protects neurons in culture from amyloid toxicity may be related to the increase in levels of tau and, ultimately, neurite stabilization and outgrowth.

Neurons in the human CNS are generally considered to be unable to regenerate, as a result of both an inhibitory environment and their inherent inability to re-grow.^33^ Importantly, it has been shown that cAMP presentation at nerve growth cones can influence their turning behavior,^34^ affect guidance^35^ and enable nerve cells from mature animals to extend fibers across inhibitory substrates.^36^ In this context, it is of interest that DNP induces an increase in cAMP levels which leads to the activation of extracellular signal regulated kinase (ERK).^28,37^ Thus, modulation of neuronal cAMP levels by DNP could also lead to promising new strategies for neuroregeneration.

Finally, it is noteworthy that the amyloid aggregation blocker and neuroprotective actions described in the previous section as well as the induction of neuritogenesis and neuronal differentiation by low concentrations of DNP described above did not involve changes in mitochondrial function in the cells in culture. No changes were observed in oxygen consumption,^28^ mitochondrial membrane potential (measured with the potential-sensitive fluorescent dye JC-1) or ROS generation (measured by DCFDA fluorescence) of N2A cells or cortical neurons treated with up to 20 βM DNP (De Felice, unpublished observations). These observations indicate that the beneficial effects of DNP discussed above are not related to mitochondrial uncoupling.

## Conclusion

The effects of DNP in the prevention of neurotoxicity and aggregation of β-amyloid, promotion of neurite outgrowth and neuronal differentiation reviewed above suggest that DNP should be explored as a lead compound for the development of novel therapeutic approaches for AD and, possibly, for other devastating and as yet incurable neurological disorders. Nevertheless, considering its well-known toxicity, it is important to emphasize that it is not our intention to propose that DNP (even at low doses) should be re-introduced into clinical practice without more research and, in particular, a re-evaluation of its toxicological profile at low doses. We believe that a compound such as DNP, simultaneously able to target amyloid aggregation and to reinforce the neuronal network, may find use in the management and treatment of AD. Moreover, mild mitochondrial uncoupling by low doses of DNP may also be of benefit in a range of neurological disorders in which excitotoxicity and oxidative stress play major roles. Finally, the ability of DNP to stimulate neurite outgrowth and neuronal differentiation may also be of interest in strategies aimed to stimulate nerve regeneration.

## References

[1] Parascandola J (1974). Dinitrophenol and bioenergetics: an historical
perspective. Mol Cell Biochem.

[2] Lipton SA, Rosenberg PA (1994). Excitatory amino acids as a final common pathway for neurologic
disorders. N Engl J Med.

[3] Mattson M (2003). Excitotoxic and excitoprotective mechanisms: abundant targets for
the prevention and treatment of neurodegenerative disorders. Neuromolecular Med.

[4] Brand MD (2000). Uncoupling to survive? The role of mitochondrial inefficiency in
ageing. Exp Gerontol.

[5] Maragos WF, Rockich KT, Dean JJ, Young KL (2003). Pre- or post-treatment with the mitochondrial uncoupler
2,4-dinitrophenol attenuates striatal quinolinate lesions. Brain Res.

[6] Lipton SA (2006). Paradigm shift in neuroprotection by NMDA receptor blockade:
memantine and beyond. Nat Rev Drug Discov.

[7] Korde AS, Sullivan PG, Maragos WF (2005a). The uncoupling agent 2,4-dinitrophenol improves mitochondrial
homeostasis following striatal quinolinic acid injections. J Neurotrauma.

[8] Korde AS, Pettigrew LC, Craddock SD, Maragos WF (2005b). The mitochondrial uncoupler 2,4-dinitrophenol attenuates tissue
damage and improves mitochondrial homeostasis following transient focal
cerebral ischemia. J Neurochem.

[9] Mattiasson G, Shamloo M, Gido G (2003). Uncoupling protein-2 prevents neuronal death and diminishes brain
dysfunction after stroke and brain trauma. Nat Med.

[10] Jin Y, McEwen ML, Nottingham SA (2004). The mitochondrial uncoupling agent 2,4-dinitrophenol improves
mitochondrial function, attenuates oxidative damage, and increases white
matter sparing in the contused spinal cord. J Neurotrauma.

[11] Bordone L, Guarente L (2005). Calorie restriction, SIRT1 and metabolism: understanding
longevity. Nat Rev Mol Cell Biol.

[12] Barros MH, Bandy B, Tahara EB, Kowaltowski AJ (2004). Higher respiratory activity decreases mitochondrial reactive
oxygen release and increases life span in Saccharomyces
cerevisiae. J Biol Chem.

[13] Padalko VI (2005). Uncoupler of oxidative phosphorylation prolongs the lifespan of
Drosophila. Biochemistry (Mosc).

[14] Ferreira ST, De Felice FG, Chapeaurouge A (2006). Metastable, partially folded states in the productive folding and
in the misfolding and amyloid aggregation of proteins. Cell Biochem Biophys.

[15] Czech C, Tremp G, Pradier L (2000). Presenilins and Alzheimer's disease: biological functions and
pathogenic mechanisms. Prog Neurobiol.

[16] Ernst RL, Hay JW, Fenn C, Tinklenberg J, Yesavage JA (1997). Cognitive function and the costs of Alzheimer's disease. An
exploratory study. Arch Neurol.

[17] De Felice FG, Ferreira ST (2002). Beta-amyloid production, aggregation, and clearance as targets
for therapy in Alzheimer's disease. Cell Mol Neurobiol.

[18] Klein WL, Stine WB Jr, Teplow DB (2004). Small assemblies of unmodified amyloid beta-protein are the
proximate neurotoxin in Alzheimer's disease Neurobiol. Aging.

[19] Ferreira ST, Vieira MNN, De Felice FG (2007). Soluble protein oligomers as emerging toxins in amyloid
diseases. IUBMB Life.

[20] De Felice FG, Houzel JC, Garcia-Abreu J (2001). Inhibition of Alzheimer's disease beta-amyloid aggregation,
neurotoxicity, and in vivo deposition by nitrophenols: implications for
Alzheimer's therapy. FASEB J.

[21] De Felice FG, Vieira MN, Saraiva LM (2004). Targeting the neurotoxic species in Alzheimer's disease:
inhibitors of Abeta oligomerization. FASEB J.

[22] Vieira MNN, Figueroa-Villar JD, Meirelles MNL, Ferreira ST, De Felice FG (2006). Small molecule inhibitors of lysozyme amyloid
aggregation. Cell Biochem Biophys.

[23] Raghu P, Reddy GB, Sivakumar B (2002). Inhibition of transthyretin amyloid fibril formation by
2,4-dinitrophenol through tetramer stabilization. Arch Biochem Biophys.

[24] Cardoso I, Merlini G, Saraiva MJ (2003). 4-Iodo-4-deoxydoxorubicin and tetracyclines disrupt transthyretin
amyloid fibrils in vitro producing noncytotoxic species: screening for TTR
fibril disrupters. FASEB J.

[25] Benitez-King G, Ramirez-Rodriguez G, Ortiz L, Meza I (2004). The neuronal cytoskeleton as a potential therapeutic target in
neurodegenerative diseases and schizophrenia. Curr Drug Targets CNS Neurol Disord.

[26] Iqbal K, Alonso Adel C, Chen S (2005). Tau pathology in Alzheimer's disease and other
tauopathies. Biochim Biophys Acta.

[27] Scheff SW, Price DA (2003). Synaptic pathology in Alzheimer's disease: a review of
ultrastructural studies. Neurobiol Aging.

[28] Wasilewska-Sampaio AP, Silveira MS, Holub O (2005). Neuritogenesis and neuronal differentiation promoted by
2,4-dinitrophenol, a novel anti-amyloidogenic compound. FASEB J.

[29] Weingarten MD, Lockwood AH, Hwo SY, Kirschner MW (1975). A protein factor essential for microtubule
assembly. Proc Natl Acad Sci USA.

[30] Drubin DG, Kirschner MW (1986). Tau protein function in living cells. J Cell Biol.

[31] Avila J, Lucas JJ, Perez M, Hernandez F (2004). Role of tau protein in both physiological and pathological
conditions. Physiol Rev.

[32] De Felice FG, Wu D, Lambert MP (2007). Alzheimer's disease-type neuronal tau hyperphosphorylation
induced by Abeta oligomers. Neurobiol Aging.

[33] Jacobs WB, Fehlings MG (2003). The molecular basis of neural regeneration. Neurosurgery.

[34] Song HJ, Ming GL, Poo MM (1997). cAMP-induced switching in turning direction of nerve growth
cones. Nature.

[35] Song H, Ming G, He Z (1998). Conversion of neuronal growth cone responses from repulsion to
attraction by cyclic nucleotides. Science.

[36] Cai D, Qiu J, Cao Z, McAtee M, Bregman BS, Filbin MT (2001). Neuronal cyclic AMP controls the developmental loss in ability of
axons to regenerate. J Neurosci.

[37] De Felice FG, Wasilewska-Sampaio AP, Barbosa ACAP, Gomes FCA, Klein WL, Ferreira ST (2007). Cyclic AMP enhancers and Ab oligomerization blockers as potential
therapeutic agents in Alzheimer's disease. Curr Alzheimer Res.

